# Importance of alpha-gal syndrome in patients undergoing catheter ablation

**DOI:** 10.1016/j.hrcr.2023.10.001

**Published:** 2023-10-25

**Authors:** Kanae Hasegawa, Edward M. Powers, Zachary T. Yoneda, Travis D. Richardson, Kara K. Siegrist, William G. Stevenson

**Affiliations:** ∗ Cardiovascular Division, Department of Medicine, Vanderbilt University Medical Center, Nashville, Tennessee; † Department of Cardiovascular Medicine, Faculty of Medical Sciences, University of Fukui, Fukui, Japan; ‡ Division of Cardiothoracic Anesthesiology, Department of Anesthesiology, Vanderbilt University Medical Center, Nashville, Tennessee

**Keywords:** Ablation, Alpha-gal syndrome, Heparin, Thrombus, Ventricular arrhythmia

## Introduction

**Key Teaching Points**

• In patients with allergy to galactose-α-1, 3-galactose (alpha-gal), there is a risk of an allergic reaction to heparin.
• Anti-alpha-gal immunoglobulin E measurement prior to procedures can be used to formulate a treatment plan including premedication in the hope of reducing the risk of heparin, should it be desired.
• A patient with alpha-gal allergy who was not initially heparinized for ablation of a right ventricular tachycardia, rapidly formed a mobile intracardiac thrombus, seen on intracardiac echocardiography, that resolved after heparin was administered.

Allergy to galactose-α-1, 3-galactose (alpha-gal) in nonprimate mammalian meat and derived products is recently recognized and increasing in frequency. Porcine-derived heparin can contain alpha-gal and allergic reactions have been reported, although data are limited. Here, during ventricular tachycardia (VT) ablation, intracardiac echocardiography (ICE) showed rapid formation of a mobile thrombus in a patient with a history of allergy to alpha-gal. An approach to management is discussed.

## Case report

**Figure 1:**
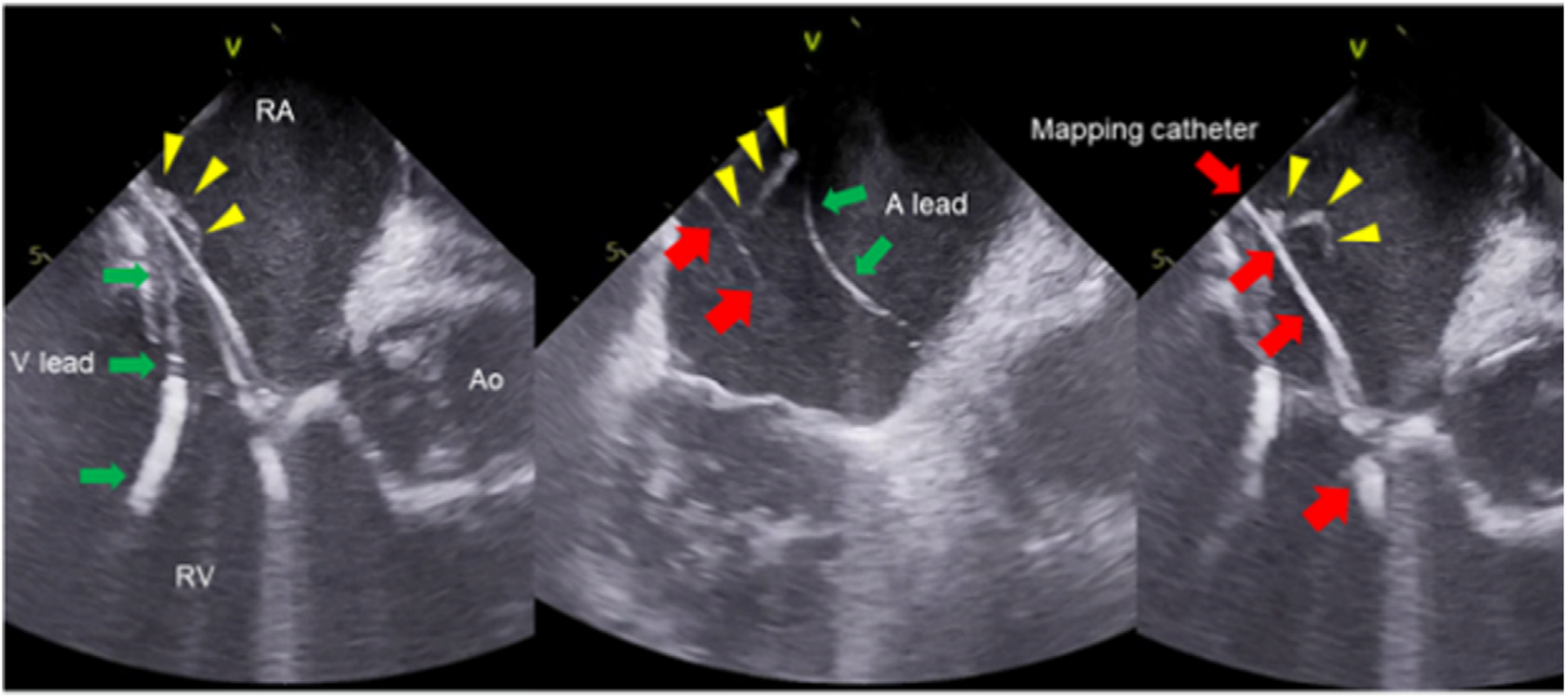
Consecutive images of the patient were recorded using intracardiac echocardiography. The yellow arrowheads indicate the thrombus. The red arrows indicate the mapping catheter. The green arrows indicate the ICD lead. A lead = atrial lead; Ao = aorta; RA = right atrium; RV = right ventricle; V lead = ventricular lead.

A 56-year-old man with repaired tetralogy of Fallot with pulmonary insufficiency, pulmonary hypertension, right ventricular (RV) dysfunction, atrial flutter, transvenous implanted defibrillator, and recurrent VT was referred for catheter ablation. He had a history of allergy to alpha-gal manifesting as rash and edema 2.5 years ago. Transthoracic echocardiography showed normal left ventricle size and left ventricular ejection fraction of 55%. The RV was severely dilated with moderately depressed systolic function and mild pulmonary stenosis. He was chronically on aspirin 81 mg and apixaban, which was held for 3 days prior to the procedure, considering the possibility that an epicardial approach may be required. Alpha-gal-specific immunoglobulin E (IgE) level was 2.84 kU/L (normal <0.1 kU/L). As heparin use was anticipated, he was premedicated following a previously reported regimen,^1^ with prednisone 50 mg at 7 and 13 hours preprocedure, cetirizine 20 mg at 13 hours, diphenhydramine 50 mg injection at 1 hour, and hydrocortisone 100 mg injection preprocedure (shown in Figure 1 of ref 1). The activated clotting time was 138 seconds (normal) before the procedure. Catheters were inserted via the femoral veins. Heparin was not initially administered owing to concern for the risk of an allergic response given the alpha-gal allergy. Rapid VT was induced and was terminated with cardioversion. An ICE catheter was used to create RV geometry and a detailed substrate map of the RV was obtained during sinus rhythm. After mapping, ICE showed a mobile linear structure (0.2 × 2 cm) on the shaft of the mapping catheter in the right atrium, consistent with thrombus (Figure 1, Supplementary Video 1). A heparin challenge dose (10 U/kg [694 U]) was administered and was tolerated without a reaction for 15 minutes. Heparin 5000 IU was then administered. Within 17 minutes after 5000 IU (full-dose) heparin, the activated clotting time was 224 seconds and thrombus was no longer visible. Radiofrequency ablation of the infundibular septal isthmus below the pulmonary valve ring was performed and abolished inducible VT. He displayed no signs or symptoms of pulmonary embolism. Apixaban was restarted and he was discharged the next day without complications. There were no episodes of VT or symptomatic embolic events over the following 3 months.

## Discussion

ICE often identifies mobile thrombi on cardiac implantable electronic device leads and occasionally catheters or sheaths at the time of ablation.^2^ Their significance is uncertain, although pulmonary emboli contributing to pulmonary hypertension has been suggested.^2^ Heparin is usually administered even for purely right-sided procedures. In this case heparin was initially held because of a history of alpha-gal allergy.^1^

Allergy to alpha-gal in nonprimate mammalian meat and derived products is only recently recognized. It occurs in subjects previously exposed to alpha-gal through a bite from the lone star tick (*Amblyomma americanum*), eliciting an IgE response. It was first identified in North Carolina and Tennessee in 2007, but the vector is now found throughout the southeastern United States.^1^ Porcine-derived heparin, gelatin, monoclonal antibodies, glycerin, and magnesium stearate can contain alpha-gal and allergic reactions have been reported, although data are limited to retrospective studies.^1^ Nwamara and colleagues^3^ reported that only 1 reaction, manifesting as a papular rash, occurred among 39 (2.6%) patients who received unfractionated heparin. Hawkins and colleagues^4^ reported that up to 50% of patients receiving heparin for cardiopulmonary bypass had reactions ranging from hives and maculopapular rash to anaphylaxis, pulmonary hypertension, wheezing, hypoxia, and vasoplegia. Moreover, anti-alpha-gal IgE levels less than 8 kU/L were suggested to be associated with a low risk of serious reaction.^4^

When ablation is undertaken in patients with the alpha-gal syndrome, the risk for allergic reactions and the benefit of intraoperative anticoagulant administration to prevent thrombus are important considerations. Though alternative parenteral anticoagulants are available (bivalirudin, argatroban), heparin remains the anticoagulant of choice given its ease of titration and rapid reversibility with protamine. A suggested approach for individuals with a history of alpha-gal allergy is to obtain alpha-gal-specific IgE levels, and to use an alternative to heparin, such as bivalirudin or argatroban if the level is high. If IgE is low, premedication with glucocorticoids and antihistamines and administration of a heparin test dose before administration of the full dose is reasonable,^1^ as was done in our case.

Because our procedure was anticipated to be confined to the venous circulation, we elected to avoid heparin administration and were surprised to observe emergence of a thrombus on the mapping catheter. Following administration of heparin, the thrombus was no longer observed and no signs or symptoms of pulmonary embolism occurred, suggesting that even if the thrombus embolized to the pulmonary circulation it likely dissolved.

## Conclusion

Alpha-gal syndrome is likely to be encountered with increasing frequency. This case demonstrates an approach to preprocedure assessment and management of the potential allergic risk. Moreover, it shows that ICE may be useful for assessing formation of thrombi that shifts the risk-benefit consideration of heparin administration for right heart procedures.

## Disclosures

All authors have nothing to disclose.

## Appendix Supplementary Data

Supplemental Video 1 Video image of the patient were recorded using intracardiac echocardiography.
